# Anak Krakatau triggers volcanic freezer in the upper troposphere

**DOI:** 10.1038/s41598-020-60465-w

**Published:** 2020-02-27

**Authors:** A. T. Prata, A. Folch, A. J. Prata, R. Biondi, H. Brenot, C. Cimarelli, S. Corradini, J. Lapierre, A. Costa

**Affiliations:** 1 Barcelona Supercomputing Center, Computer Applications in Science and Engineering, Barcelona, Spain; 2 AIRES Pty. Ltd., Mt Eliza, Victoria, Australia; 3 Visiting Professor, School of Electrical Engineering, Computing and Mathematical Sciences, Curtin University, Perth, Australia; 4 Università degli Studi di Padova, Dipartimento di Geoscienze, Padua, Italy; 5 Royal Belgian Institute for Space Aeronomy, Brussels, Belgium; 6 Department of Earth and Environmental Sciences, Ludwig-Maximilians-Universität, München, Germany; 7 Istituto Nazionale di Geofisica e Vulcanologia, Osservatorio Nazionale Terremoti, Rome, Italy; 8 Earth Networks Inc., Germantown, MD United States; 9 Istituto Nazionale di Geofisica e Vulcanologia Sezione di Bologna, Bologna, Italy

**Keywords:** Natural hazards, Volcanology

## Abstract

Volcanic activity occurring in tropical moist atmospheres can promote deep convection and trigger volcanic thunderstorms. These phenomena, however, are rarely observed to last continuously for more than a day and so insights into the dynamics, microphysics and electrification processes are limited. Here we present a multidisciplinary study on an extreme case, where volcanically-triggered deep convection lasted for six days. We show that this unprecedented event was caused and sustained by phreatomagmatic activity at Anak Krakatau volcano, Indonesia during 22–28 December 2018. Our modelling suggests an ice mass flow rate of ~5 × 10^6^ kg/s for the initial explosive eruption associated with a flank collapse. Following the flank collapse, a deep convective cloud column formed over the volcano and acted as a ‘volcanic freezer’ containing ~3 × 10^9^ kg of ice on average with maxima reaching ~10^10^ kg. Our satellite analyses reveal that the convective anvil cloud, reaching 16–18 km above sea level, was ice-rich and ash-poor. Cloud-top temperatures hovered around −80 °C and ice particles produced in the anvil were notably small (effective radii ~20 µm). Our analyses indicate that vigorous updrafts (>50 m/s) and prodigious ice production explain the impressive number of lightning flashes (~100,000) recorded near the volcano from 22 to 28 December 2018. Our results, together with the unique dataset we have compiled, show that lightning flash rates were strongly correlated (R = 0.77) with satellite-derived plume heights for this event.

## Introduction

Tropical thunderstorms can be triggered in a variety of ways. Common triggering mechanisms include solar heating, convergence of surface winds and the flow of wind over topography^1^. A less studied mechanism is in the case of an erupting volcano where the input of heat at the surface initiates deep convection^2–4^. Intense heating at ground surface and entrainment of moist air generates positive buoyancy^5^, which rapidly transports volcanic gases and ash particles up to the tropopause and beyond. Here we present the first detailed account of tropical deep convection triggered and sustained by magma-seawater interactions at an island volcano.

**Figure 1:**
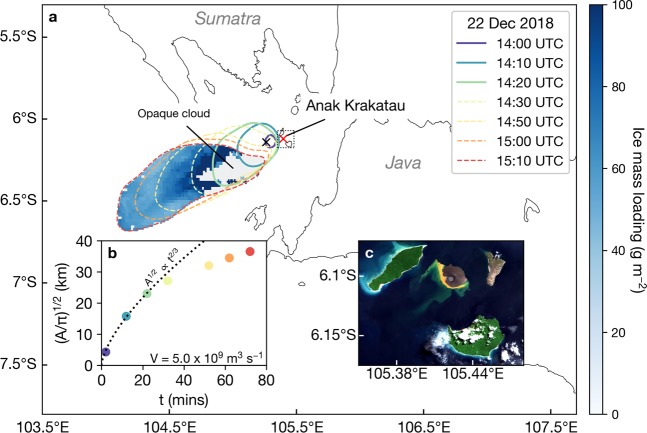
Umbrella spread analysis of the initial explosive phase of the Anak Krakatau eruption. (**a**) Umbrella spread of the initial plume with Himawari-8 ice mass loading retrievals (blue shading) plotted underneath. Coloured contours indicate Himawari-8 11.2 μm brightness temperature isotherms (245 K with gaussian filter) at 10 minute intervals. Dashed contours indicate times when the plume is considered to be detached. The red ‘x’ indicates parallax corrected location of the minimum cloud-top temperature at 14:00 UTC (black ‘x’ denotes uncorrected location ~15 km from the volcano). Note that 14:40 UTC is a ‘house-keeping’ time (data are not recorded by the Himawari-8 sensor at this time). Ice mass retrievals are only shown for pixels inside the isoline at 15:10 UTC. (**b**) Change in average radius with time (t) in minutes after the plume begins spreading at the NBL (inferred to be 13:58 UTC on 22 December 2018). Dashed line shows a non-linear least-squares fit to the first three data points (when the plume is considered to be feeding the umbrella cloud) using the Woods and Kienle model^45^ (*V* is the volumetric flow rate). Coloured dots correspond to observation times shown in the legend. (**c**) Atmospherically-corrected (using the Sen2Cor algorithm^81^) true colour Sentinel-2 image (02:56 UTC, 31 March 2019) showing the remnant edifice of Anak Krakatau after the sustained convective eruption. Maps and satellite imagery were generated and processed by the authors using Matplotlib 3.0.3^82^ and Python 3.6.7 (https://www.python.org/).

Anak Krakatau (‘Child of Krakatau’) is an island volcano located in Indonesia’s Sunda Strait (6 °06′07″S, 105 °25′23″E) between the islands of Java and Sumatra (Fig. 1). The volcano first appeared in January 1927 having formed in the caldera left behind by the famous cataclysmic eruption of Krakatau in 1883^6^. On 22 December 2018, Anak Krakatau underwent a major explosive eruption after experiencing six months of intense Strombolian to Vulcanian activity. The eruption resulted in a flank collapse on the southwestern side of the volcano^7,8^, which generated a deadly tsunami that hit the coasts of Java and Sumatra at 21:27 LT (14:27 UTC)^9^. The flank collapse marked the beginning of sustained phreatomagmatic activity at the volcano and led to the formation of a deep convective plume recorded by satellite for about six days.

### Initial explosive event

We analysed the eruption sequence in great detail using geostationary and polar orbiting satellite data. The initial eruption can be identified in the thermal infrared data as a rapidly expanding cloud reaching cloud-top temperatures of −80 °C before detaching from the volcano (at around 14:30 UTC) and advecting to the southwest (Fig. 1a). Our umbrella spread analysis (see Methods) reveals that the plume expanded as a gravity current around its neutral buoyancy level (NBL) and was fed by an average volumetric flow rate of 5 × 10^9^ m^3^/s (Fig. 1b). The initial volcanic cloud did not exhibit the typical ‘reverse absorption’ signature observed for volcanic ash clouds in infrared satellite data (i.e. highly negative 11.2–12.4 μm brightness temperature differences)^10^, but produced highly positive 8.6–11.2 μm brightness temperature differences (up to 9 K) indicative of an ice-rich cloud composed of small particles^11,12^.

**Figure 2:**
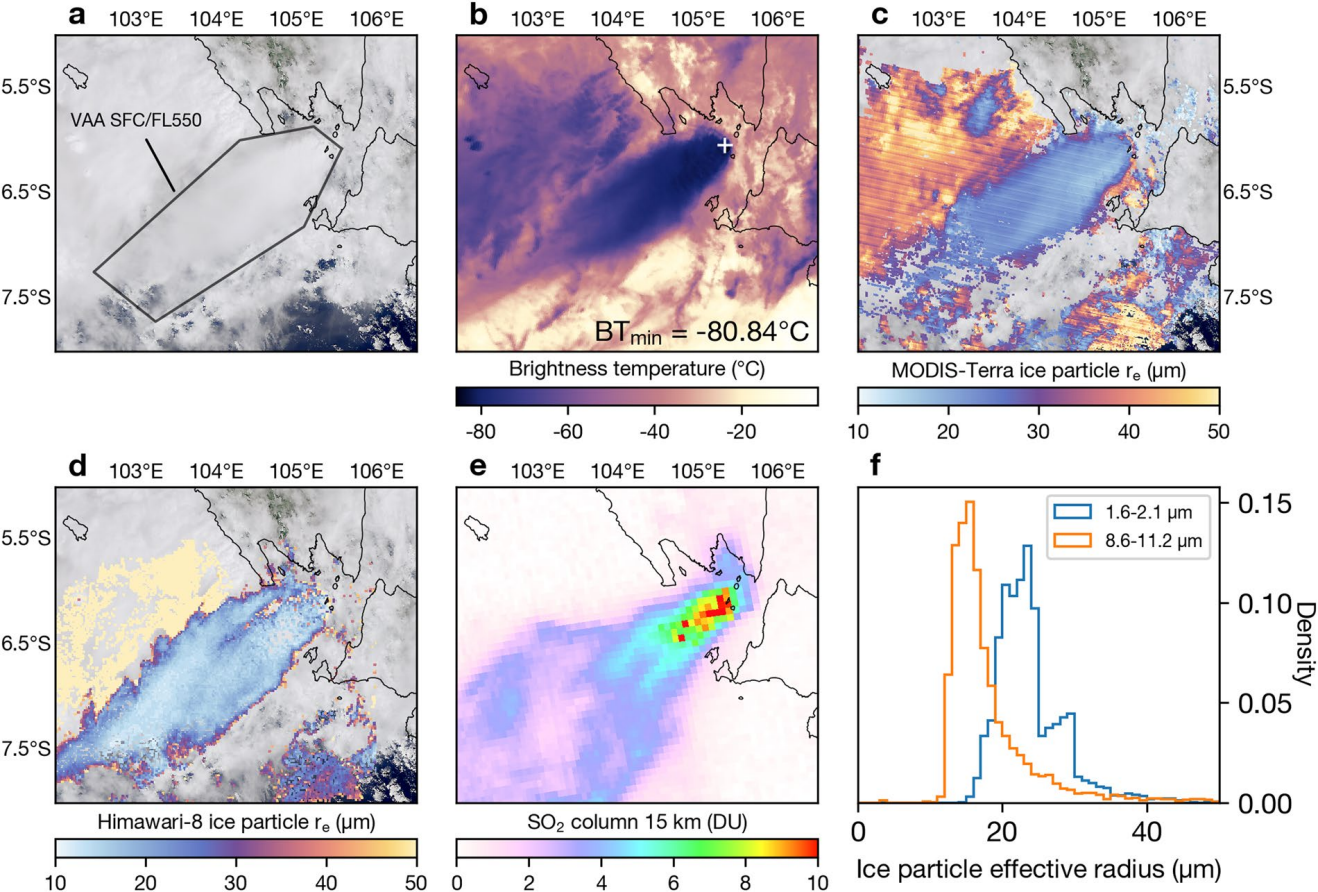
Anatomy of the Anak Krakatau convective anvil plume. (**a**) Atmospherically-corrected true colour MODIS-Terra observation at 03:05 UTC on 23 December 2018 (processed using the SatPy package^83^). Darwin VAAC (Volcanic Ash Advisory Center) volcanic ash advisory (VAA) from surface (SFC) to flight level 550 (FL550; 55,000 ft) at 02:45 UTC on 23 December is over-plotted. (**b**) Same as (**a**) but for MODIS-Terra 11.0 μm channel. (**c**) Same as (**b**) but for the level 2 cloud product (MOD06) of ice particle effective radius using the combined 1.6 and 2.1 μm retrieval. (**d**) Himawari-8 thermal infrared ice particle effective radius retrieval using channels 8.6 and 11 μm (see Methods) at 03:00 UTC on 23 December 2018 (with MODIS true colour under-plotted). (**e**) TROPOMI SO_2_ retrieval (product shown is for a 1 km box vertical profile centred on 15 km asl and 7 × 3.5 km^2^ horizontal resolution^84^) at 07:05 UTC on 23 December 2018. (**f**) Histograms of the 1.6–2.1 μm (MODIS) and 8.6–11.2 μm (Himawari-8) retrievals shown in (**c**,**d**) respectively. All histograms correspond to retrievals inside the VAA polygon (shown in **a**). Maps and satellite imagery were generated and processed by the authors using Matplotlib 3.0.3^82^ and Python 3.6.7 (https://www.python.org/).

**Figure 3:**
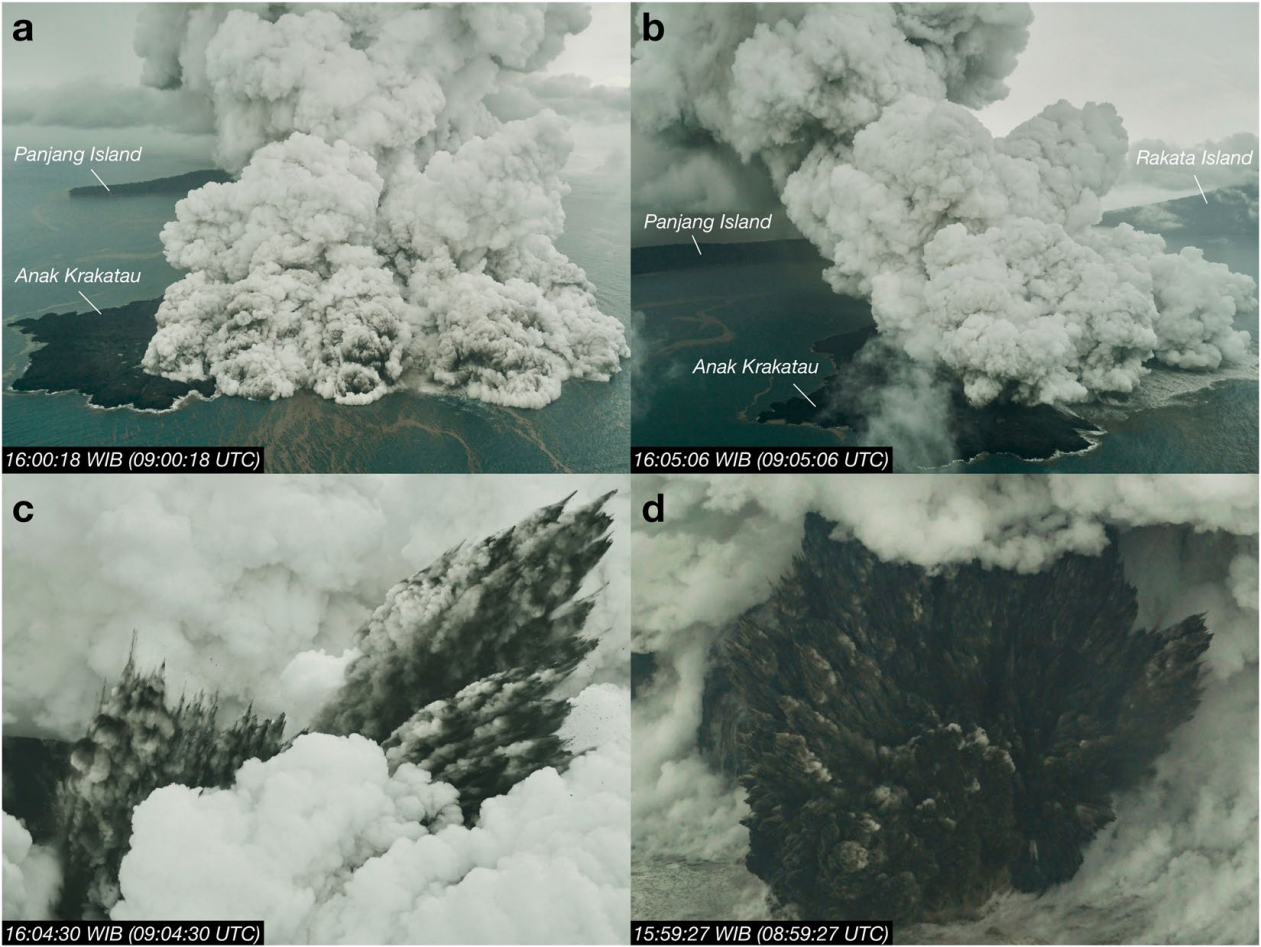
Aerial photographs of the Anak Krakatau eruption on 23 December 2018. (**a**,**b**) show two angles of the sprawling phreatomagmatic cloud. Plume extends toward the east over Panjang Island. (**c**,**d**) Cock’s tail jets characteristic of Surtseyan volcanic activity. All photographs used with permission from the copyright owner Dicky Adam Sidiq/kumparan.

Following the initial explosive event, a highly electrified convective plume resembling a tropical anvil cloud began to form (Fig. 2a,b; Supplementary Video 1). A sequence of aerial photographs taken by a journalist on board a Susi Air Cessna aircraft on 23 December shows a sprawling phreatomagmatic cloud (Fig. 3a,b), dark cock’s tail jets of ash (Fig. 3c,d) and a white plume extending toward the east over Panjang Island. These observations are reminiscent of the eruptions of Surtsey in Iceland in 1964^13,14^. The availability of salt and moisture at the surface, provided by evaporated seawater and a tropical moist atmosphere, suggest that most of the ash remaining in the plume after sedimentation was efficiently removed from the atmosphere at low levels via wet deposition and aggregation^15,16^. Low level winds were predominantly toward the east and northeast from 22 to 28 December 2018 and evidence of ash deposition on Panjang Island (east of Anak Krakatau) is clearly seen in the Senintel-2 true colour image on 31 March 2019 (Fig. 1c). At higher altitudes, where prevailing winds were towards the west and southwest, any fine ash particles remaining in the plume were coated with ice, hiding their spectral signature from satellite ash detection algorithms (Fig. 2c,d).

### Time series analyses

**Figure 4:**
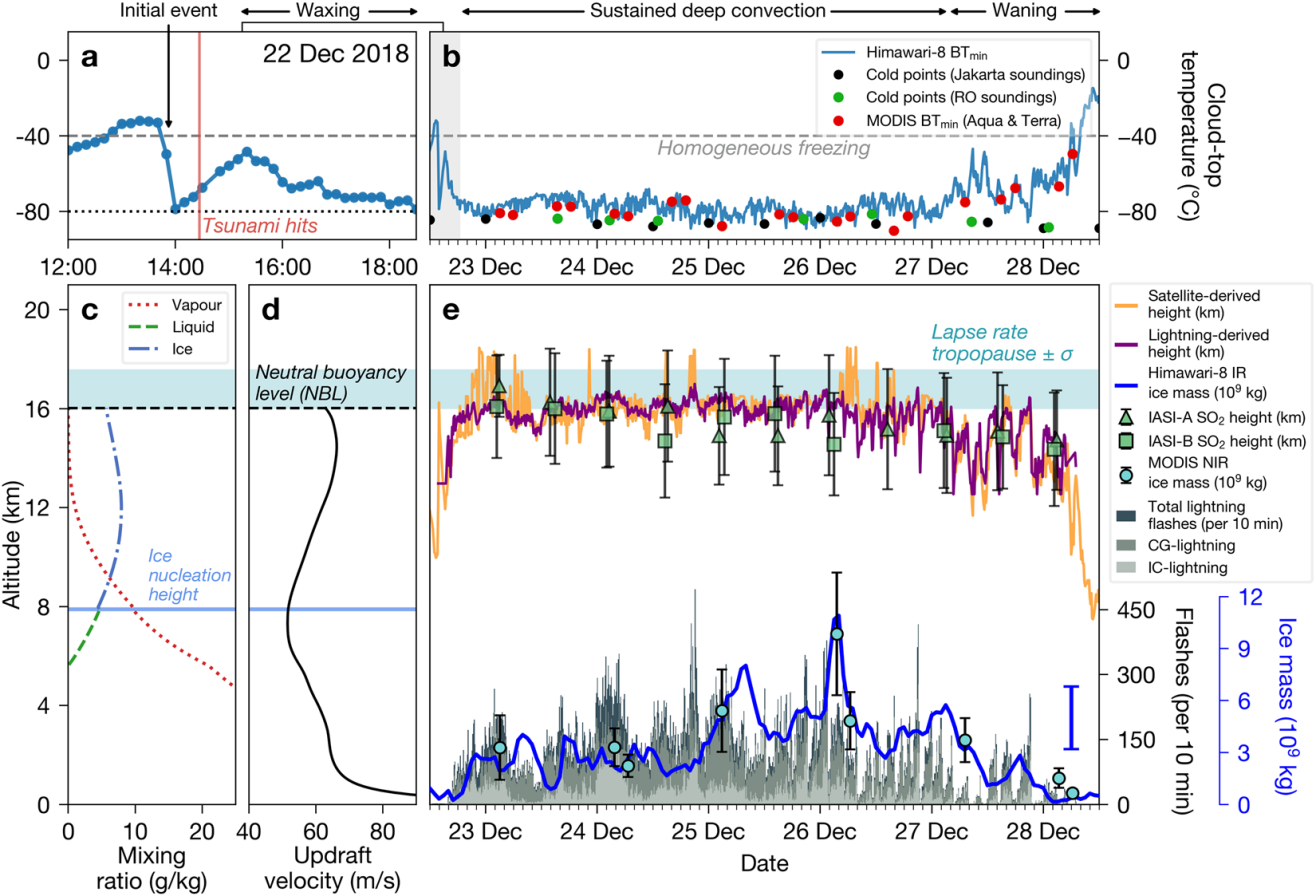
Time series of the Anak Krakatau convective plume. (**a**) Himawari-8 11.2 μm minimum brightness temperatures (blue line with dots) from 12:00–18:30 UTC (22 December 2018) in a 0.25° × 0.25° latitude/longitude box centred over Anak Krakatau. (**b**) Same as (**a**) but from 12:00 UTC 22 December 2018 to 12:00 UTC 28 December 2018. Red dots indicate MODIS minimum brightness temperatures. Black and green dots indicate minimum temperatures from Jakarta airport (6.11 °S, 106.65 °E; accessed from http://weather.uwyo.edu/upperair/) and Radio Occultation (RO) soundings, respectively. (**c**) FPLUME modelled water vapour, liquid and ice mass mixing ratio profiles with NBL (see Methods for definition) plotted as dashed line (also shown on **d**). ERA5 atmospheric profile at 12:00 UTC on 22 December 2018 was used in the model. Shaded horizontal area indicates lapse rate tropopause ± standard deviation (*σ*) according to ERA5 data and RO soundings (also shown on **d** and **e**). (**d**) FPLUME updraft velocity profile (shares left axis with **c**). (**e**) Plume height time series. Purple line indicates heights estimated using total flash rate at 10 minute intervals. Orange line indicates heights derived from Himawari-8. Green triangles and squares indicate retrieved SO_2_ heights from IASI-A and B, respectively. All heights correspond to left axis of (**c**). Bottom grey shaded histograms indicate flash rates (CG = cloud-to-ground and IC = intracloud; right black axis; black y-label). Blue line indicates ice mass loadings derived from Himawari-8 at hourly intervals (mean retrieval error, ±1.8 × 10^9^ kg, indicated by floating blue error bar). Circles with error bars indicate ice mass loadings from MODIS (Aqua and Terra) retrieved from NIR measurements (right blue axis; blue y-label).

To unpack the sequence of events during and following the flank collapse, we compiled a unique time series dataset, combining polar orbiting and geostationary satellite data with lightning data from the Earth Networks Global Lightning Networks (ENGLN). The time series, shown in Fig. 4, covers the period from 12:00 UTC on 22 December 2018 to 12:00 UTC on 28 December 2018. The initial explosive event appears in the time series as a sharp drop in brightness temperature at 13:50 UTC on 22 December, 37 minutes before the tsunami hit the coasts of Java and Sumatra (Fig. 4a). By 14:00 UTC, brightness temperatures had reached −80 °C, suggesting a rapid vertical ascent of the plume. At around 15:20–15:30 UTC the convective anvil began to develop, reaching maturity by 18:30 UTC (Fig. 4a). To estimate the height of the plume, we matched satellite-measured brightness temperatures (Fig. 4b) to temperatures in ERA5 meteorological profiles over the volcano and extracted the corresponding heights (see Methods). Remarkably, the plume maintained heights of 16 km continuously for almost five days, occasionally reaching heights of 18 km (Fig. 4d; left axis). These heights are in agreement with a feature detected by independent space-borne lidar at 16–17 km on 24 December at 18:50 UTC (Supplementary Fig. 1)^17^ and are larger than the maximum height reached by previous magma-seawater eruptions (cf. Capelinhos^18^, Surtsey^13^ and Bogoslof^19^ eruptions, 4, 9 and 14 km, respectively). Analysis of satellite radio occultation sounding data (see Methods) indicates that the mean lapse rate tropopause for December was 16.8 km ± 0.8 km (green shaded area; Fig. 4c–e). Thus the height of the tropopause played a major role in controlling the height of the convective plume^20^.

From 22 to 31 December the ENGLN network recorded an incredible 102,676 lightning flashes (cloud-to-ground plus intracloud), equating to an average flash rate of 8.7 min^−1^ and a maximum of 72 min^−1^ (using a 1-minute average) at 21:05 UTC on 24 December. Flash rates greater than 32.9 min^−1^ occur in less than 0.1% of meteorological thunderstorms^21^. We used the ENGLN data to derive an empirical parameterization, correlating lightning flash rate (per 10 min) with Himawari-8 cloud-top heights (see Methods). For the first time for this kind of event, we show that there is a strong correlation (*R* = 0.77, *p*-value < 0.001; Supplementary Fig. 2) between lightning flash rates and satellite-derived plume heights (compare purple line to orange line on Fig. 4e).

### Plume dynamics, microphysics and electrification

To gain insight into the dynamics and microphysics of the initial explosive eruption on 22 December, we performed model simulations (see Methods) using the 1D volcanic plume model FPLUME^22^. We initialised the model with 0–50 wt% water, noting that water contents ≥40% led to column collapse. Liquid water mass fractions at the vent from 0–30 wt% were capable of sustaining a convective column with a neutral buoyancy level (NBL) at ~16 km (Supplementary Fig. 5). For a water content of 20 wt%, the model simulations reveal that water vapour and liquid water exist between 5 and 8 km and a mixed-phase layer (water vapour and ice) exists from 8–16 km with ice nucleation beginning at around 8 km (Fig. 4c). The 20 wt% simulation indicates an ice mass flow rate of 4.8 × 10^6^ kg/s and an ice-air mixture volumetric flow rate of 4.2 × 10^9^ m^3^/s at the NBL. The modelled volumetric flow rate is in very good agreement with our independent satellite-based estimate (5 × 10^9^ m^3^/s; Fig. 1a,b). Our simulations also suggest mean updraft velocities of ~60 m/s (Fig. 4d), which implies that the plume travelled 16 km in ~4 minutes. Based on our umbrella analysis, the plume began spreading at the NBL at ~13:58 UTC (Fig. 1; Methods), which suggests that it began its vertical ascent at ~13:54 UTC. These inferences are plausible considering that the flank collapse was estimated to have occurred at around 13:55 UTC^8^. This interpretation, however, does not explain the cold pixel detected by Himawari-8 at 13:50 UTC (Fig. 4a). It is possible that this anomaly was due to a rising plume associated with precursory activity just before the flank collapse.

During the period of sustained convection, satellite retrievals show that on average over six days ~3 × 10^9^ kg of ice was being maintained by the convective activity (Fig. 4e; Supplementary Fig. 3). This mass is five times the amount produced by non-volcanic convective clouds in the same vicinity (Supplementary Fig. 3) and the same order of magnitude to that produced by the eruptions of Rabaul (Papua New Guinea, 1994; 2 × 10^9^ kg)^11^ and Hekla (Iceland, 2000; 1 × 10^9^ kg)^23^. The lack of ash and abundance of ice detected during this period, coupled with significant amounts of lightning, indicates that strong upward ice mass fluxes played a major role in the charge separation required to generate the lightning^24^. We speculate that non-inductive charging, generated by collisions between updrafts of small ice crystals and larger falling graupel^25,26^, was the main charging mechanism—a mechanism commonly attributed to lightning in meteorological thunderstorms^27^, but also previously invoked to explain charging mechanisms in volcanic thunderstorms^28,29^.

### Sustaining mechanisms

**Figure 5:**
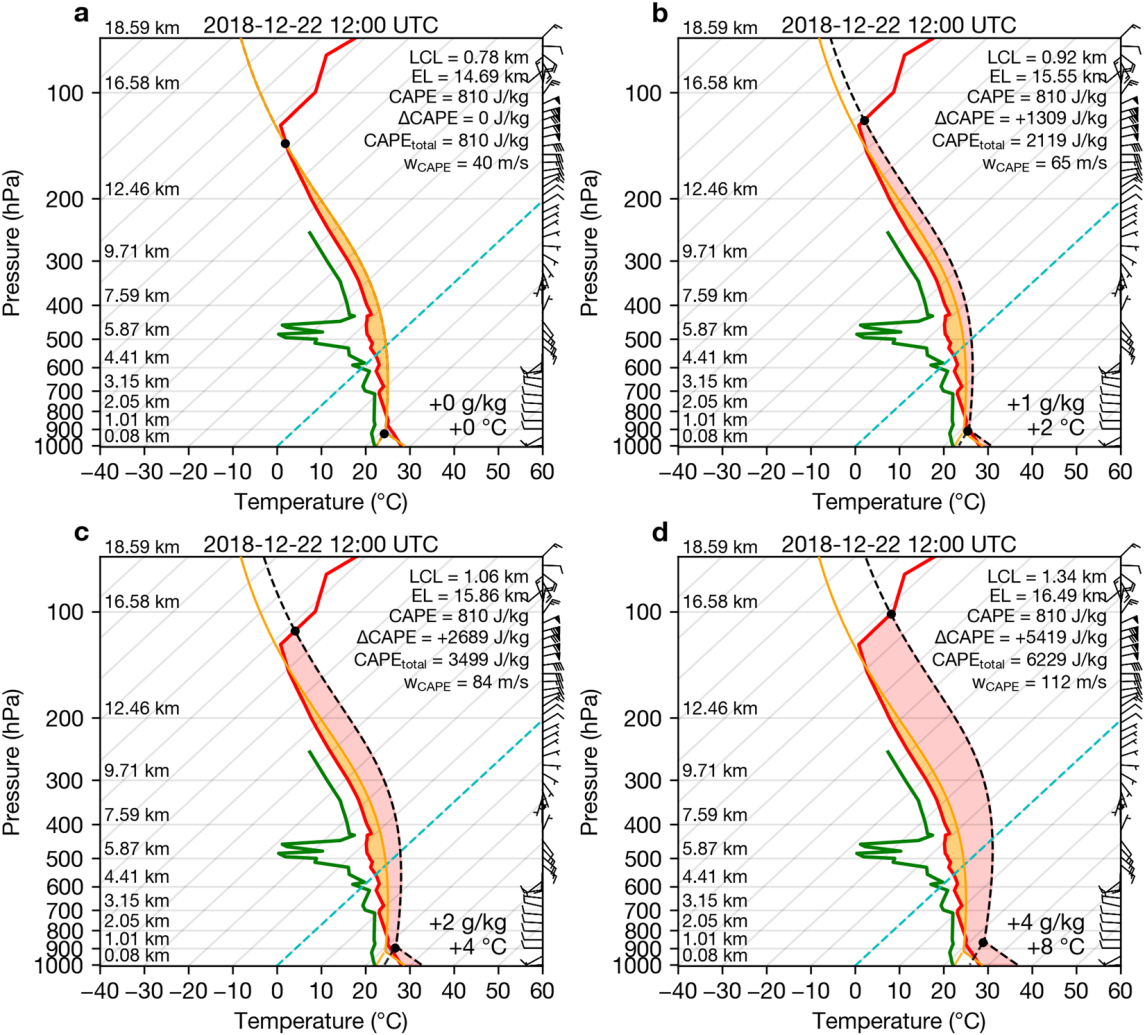
Thermodynamic skew-T diagrams for increases in surface parcel temperature and moisture. Red and green lines indicate environmental temperature and dew point temperature, respectively, for a Jakarta airport sounding at 12:00 UTC on 22 December 2018 (accessed from: http://weather.uwyo.edu/upperair/). Orange solid lines indicate environmental parcel profiles (CAPE shaded in orange, Convective inhibition shaded in blue). Dashed black lines indicate theoretical parcel profiles for surface parcels with varying amounts of added heat and moisture (**a**–**d**). Lower and upper black dots indicate the lifted condensation level (LCL) and equilibrium level (EL), respectively. Red shaded area indicates increases in CAPE due to added surface heat and moisture (i.e. volcanic-CAPE). Maximum theoretical updraft velocities (*w*_*CAPE*_) are also annotated on each panel. Wind barbs shown on right side of each panel indicate wind speed and direction.

Figure 5a illustrates the thermodynamic state of the atmosphere prior to the sustained convective period. The surface-based convective available potential energy (CAPE) is ~810 J/kg, which is not large enough to support deep convection. However, if we consider that magma-seawater interactions at the surface had the effect of adding heat and moisture to surface air parcels, we find that CAPE increases significantly (+5400 J/kg) with only modest increases in parcel temperature (+8 °C) and moisture (+4 g/kg; Fig. 4b,c, d). This treatment of modified CAPE is analogous to that used for the analysis of deep convection triggered by large fires^30–32^. By considering volcanically-induced CAPE (‘volcanic-CAPE’), we find that a continual input of heat and moisture at the surface would lead to sustained, extreme convection (CAPE ~5000–6000 J/kg), but without this input, deep convection would not be expected. For CAPE of these magnitudes, parcel theory predicts maximum updrafts (${w}_{CAPE}=\sqrt{2\times CAPE}$) of the order of ~100 m/s. In reality, peak updraft velocities are generally a factor of two lower (~50 m/s) due to the effects of entrainment, water loading and perturbed vertical pressure gradients^33,34^. These updraft velocities, however, are still well above the peak updraft velocities required for strong electrification (> 10–12 m/s)^35^.

### Small ice particles

Lightning activity in thunderstorms is also associated with the occurrence of small ice particles at cloud-top^36^. We find median ice particle effective radii of 16 and 23 µm, based on retrievals (see Methods) from Himawari-8 and MODIS, respectively, in the sustained anvil cloud shown in Fig. 2c,d,f. These particle sizes are much smaller than those detected in meteorological clouds to the north and west of the plume (Fig. 2b,c,d). Cold cloud-tops (−80 °C) and strong updrafts suggest that the small ice particles were formed via a homogeneous freezing process^36–39^. Under these conditions there is no need to consider nucleation by ash seeding in order to generate ice.

In addition to strong updrafts and cold cloud-tops, the presence of sulphate aerosols has been proposed as a mechanism for producing small particles in anvil cirrus clouds^38^. Data collected from the Support to Aviation Control Service (SACS) near real-time warning system (see Methods) indicate the presence of SO_2_ during our analysis period (Fig. 4e) and so the impact of sulphate aerosols on ice particle size in the plume cannot be ruled out. The maximum total mass of SO_2_ in the upper troposphere-lower stratosphere (UTLS) was relatively minor (56 kt ± 9 kt; Supplementary Fig. 4) given the strength of the eruption, suggesting that much of the SO_2_ was scavenged by ice^11,40,41^. Although SO_2_ was detected in the lower stratosphere, these amounts are not sufficient to cause a climate impact^42^.

### Significance of the Anak Krakatau thunderstorm

Our analysis of the Anak Krakatau eruption is the first detailed account of tropical deep convection triggered and sustained by magma-seawater interactions. For the period from 22 to 28 December 2018, we have identified four phases: an initial explosive phase associated with the flank collapse, a waxing phase where convective development led to the formation of an anvil cloud, a ‘volcanic thunderstorm’ phase characterised by sustained deep convection and numerous lightning flashes and finally a waning phase where convection over the volcano eventually began to subside. Convective initiation was triggered by magma-seawater interactions at the surface and a conditionally unstable atmosphere promoted deep convection. The continual source of heat and moisture at the surface led to a sustained, positively buoyant plume extending up to the tropopause. This process led to sustained ice production in the upper troposphere.

The Anak Krakatau thunderstorm represents an end-member on the spectrum of volcanic thunderstorms owing to the copious amount of ice detected and lack of a spectral ash signature. Our time series (Fig. 4) provides convincing evidence that lighting data can be reliably used to estimate cloud-top heights in ash-poor, ice-rich volcanic thunderstorms. Our findings reveal that tropical deep convection triggered by magma-seawater interactions can lead to a sustained anvil cirrus plume packed with small ice particles. High ice water content environments are recognised as a severe aviation hazard that can lead to jet engine power loss^43^.

## Methods

### Cloud-top heights from infrared satellite data

To estimate Anak Krakatau’s cloud-top heights we took the minimum brightness temperature (one pixel) measured from the 11.2 μm channel within a 0.25° × 0.25° latitude/longitude box centered over the volcano. The minimum brightness temperature time series was then compared to ERA5 reanalysis^44^ atmospheric profiles over the volcano (137 model levels; 0.3–0.5 km vertical resolution in the UTLS) to retrieve the closest temperature (and thus height) to minimum brightness temperature measured by the satellite. This method assumes that the cloud is at thermal equilibrium with the ambient atmosphere. We estimate an error in height by this method to be ± 1 km, allowing for errors in temperature of 2 K and 3 K for Himawari-8 measurements and ERA5 profiles, respectively, and a lapse rate of ~5 K/km near the tropopause.

### Umbrella spread analysis

We used the gravity current model of Woods and Kienle^45^ that describes the change in radius with time of a spreading umbrella cloud at the neutral buoyancy level (NBL). The volumetric flow rate *V* (in m^3^/s) of the initial Anak Krakatau cloud on 22 December 2018 is computed by applying a non-linear least squares fit of the following equation to estimates of the plume radius at each Himawari-8 observation:

$$R={(\frac{3\lambda NV}{2\pi })}^{1/3}{t}^{2/3}$$

where *R* is the radius of the plume, *λ* is an empirical constant (assumed here to be 0.2), *N* is the Brunt Väisälä frequency (calculated to be 0.015) and *t* is the time since the plume begins spreading at the NBL (inferred to be 13:58 UTC on 22 December 2018). Only data points that correspond to times where the plume is activity feeding the umbrella are considered in the non-linear least squares fit (i.e. first three data points shown in Fig. 1c). Note that we treat the radius as an average radius as the plume’s area is not exactly circular:

$$R={(A/\pi )}^{1/2}.$$

To calculate the area, *A*, we use Green’s theorem:

$$A={\iint }_{A}dxdy={\oint }_{C}xdy.$$

To obtain the necessary coordinates of the enclosed loop, *C*, around the plume at each observation, we smooth the 11.2 μm brightness temperatures using a Gaussian filter (*σ* = 3) and take the 245 K isotherm. The uncertainty in the area using this method depends on the density of the coordinate pairs (1 pixel per pair) and the geolocation accuracy of Himawari-8. According to Takeuchi^46^ the geolocation accuracy is ~1 pixel or 2 km for the IR channels. For a perfectly circular umbrella cloud the resulting error is about 10% for a cloud of radius 30 km.

### Volcanic plume modeling

We used FPLUME-1.2^22^ to model the initial explosive eruption associated with the flank collapse, as a phreatomagmatic eruption assuming water mass fractions ranging from 10 to 30 wt%, a magmatic water fraction of 5% and a magma temperature of 1150 °C (representative of basaltic-andesitic composition^47^), noting that incandescence was observed at the onset of the eruption (Supplementary Fig. 5; Supplementary Table 1). We also carried out simulations using water fractions of 40 wt% and 50 wt% but these simulations resulted in column collapse. For the 20 wt% water mass fraction simulation, the plume temperature instantaneously drops to ca. 500 °C after magma-water interaction (assuming an initial seawater temperature of 20 °C) and resulting vapor generation. We selected a representative atmospheric column at 12:00 UTC on 22 December from ERA5 to account for wind coupling, air moisture entrainment and latent heat effects assuming an exit velocity of 150 m/s and typical values of 0.12 and 0.3 for the entrainment coefficients^48^. The FPLUME model outputs vertical profiles of mixture upwards velocity and temperature, as well as mass flow rates and mass fractions for each water phase (vapor, liquid and ice). To constrain our model simulations with satellite observations, we varied the mass flow rate at the vent until the NBL from the model matched our satellite-based estimate (~16 km). The NBL is defined in FPLUME as the height at which the in-plume density equals the ambient air density. For the 20 wt% simulation, the resulting ice mass flow rate at the NBL was found to be 4.8 × 10^6^ kg/s with a total mass flow rate of 8.4 × 10^8^ kg/s. This mass flow rate corresponds to a total volumetric flow rate of 4.2 × 10^9^ m^3^/s, assuming an ice-air mixture density at the NBL of 0.2 kg/m^3^.

### Ice mass retrievals

Infrared (IR) satellite retrievals of the ice mass loading (g m^−2^) were performed using the Himawari-8^49^ channels with central wavelengths (*λ*) of 8.6 and 11.2 μm. The refractive index of ice has a strong variation at these two wavelengths^50^ with much greater absorption at 11.2 μm compared to that at 8.6 μm. Thus ice clouds appear colder at 11.2 μm and the brightness temperature difference (Δ*T*) between these two channels for the same pixel is an indication of the effect of ice particles. The amplitude of Δ*T* varies with the 8.6 μm brightness temperature of the cloud and is a function of both the cloud optical depth and ice particle microphysics (shape, size and size distribution). A model was developed to retrieve the IR optical depth and effective radius of ice particles for an assumed shape and size distribution. The required inputs to the model are the extinction efficiency (*Q*_*ext*_), the single-scatter albedo ($\varpi$) and the asymmetry parameter (*g*). These were obtained from T-matrix calculations using the extended-precision code of Mishchenko^51^. The T-matrix code itself requires inputs about the shape and size distribution of the particles. The size distribution of ice in clouds has been the subject of numerous research experiments^52–56^ and likewise the shapes of ice particles have been measured and studied for many years^57–61^. Ice particles are found as roughened spheroids, bullet rosettes, hexagonal columns and plates and aggregates with complex morphology. Generally smaller and colder ice particles are more spheroidal. The size distributions of ice particles are often found to be bimodal^62,63^ and approximated by power laws^64^ or a modified-gamma distribution^65^. For the Anak Krakatau ice clouds studied here there are no *in situ* data on the sizes or shapes of the particles. Inspection of the Δ*T* curves (plots of Δ*T* vs *T*_8.6_ brightness temperatures) suggest the particles are small (effective radii <50 μm) and cloud-top temperatures of <200 K are found. These inferences suggest that the scattering calculations must be performed in the so-called transition region where the size parameter, *x* = 2*πr*/*λ* ~ *O*(1). For mono-dispersed spheres there are exact codes based on Mie theory that provide estimates of *Q*_*ext*_, $\varpi$ and *g* for *x* ~ 0.1 to >100. For more complex shapes and size distributions approximate numerical schemes have been developed^51,66–68^. A sensitivity study was performed to assess the effects of shape and size distributions on the calculated scattering parameters. Size distribution tends to smooth out the ripples found for *x* > 1 and suppresses the amplitude of the extinction peak. Prolate, oblate and Chebyshev-shaped particles of increasing order also tend to diminish the ripples but have a smaller effect on the extinction peak. As the radius increases above ~30 μm the extinction efficiency tends towards a constant value for all shapes and size distributions.

Based on these insights, calculations were performed for spheroids with *A*/*B* = 2 within a power law size distribution for particles with equal-volume-sphere effective radii (*r*_*e*_) varying from 0.1 μm to 50 μm. Refractive indices for the 8.6 and 11.2 μm channels were calculated using the data of Warren and Brandt^50^ after integration over the Himawari-8 filter response functions. The computed scattering parameters were then supplied to a discrete ordinates method^69^ (DOM) radiative transfer program with 16 gaussian angles, infrared optical depths *τ* varying from 0.1 to 10 in steps of 0.1 with assumed cloud-top (*T*_*c*_) and surface temperatures (*T*_*s*_) of 190 to 220 K in steps of 5 K and 290 to 298 K in steps of 2 K, respectively. The outputs of the DOM code are look-up tables (LUTs) of top-of-the-atmosphere brightness temperatures at 8.6 and 11.2 μm for a range of *r*_*e*_, *τ*, *T*_*c*_, *T*_*s*_ and 16 viewing angles. Selection of the appropriate LUT is based on estimating *T*_*s*_ and *T*_*c*_ through inspection of the Himawari-8 IR data and noting that the geostationary view of the scene from Himawari-8 is at a constant view angle. Interpolation of the LUTs was done by finding polynomial fits to the Δ*T vs T*_8.6_ curves for each of the modelled effective radii. Data points found lying between two consecutive radii curves were assigned radii weighted by their distance from each curve. A similar process was used to estimate the optical depth based on curve fits. Generally, the fits are excellent. The effective radii of data points lying beyond the largest radius modelled (50 μm), and with Δ*T* above 1 K were weighted between 50 and 200 μm—the value of 200 μm was taken as an upper limit to the size range. Only data points in the region with *T*_8.6_<240 K were included based on the premise that these are likely to represent particles in the ice phase.

Himawari-8 data were acquired at 10 minute intervals and processed for the period 22 December 2018 to 28 December 2018. The *T*_8.6_ and *T*_11.2_ brightness temperatures were calculated and a water vapour correction applied to the Δ*T* based on Yu *et al*.^70^. Finally, retrievals were made by interpolating the appropriate LUT using the measurements. Retrievals that satisfied both of these conditions were rejected:

$$\Delta \,T < 1.0\,{\rm{K}}\,{\rm{and}}\,{T}_{8.6} > 240\,{\rm{K}}$$

The purpose of these tests was to reject semi-transparent clouds with little absorption difference but allow opaque clouds which haveΔ*T* ~ 1 K. A value of 240 K was assumed based on the expectation that hydrometeors in clouds with cloud-top temperatures <−40 °C would be in the ice phase.

Ice mass loading (*m*) was calculated on a pixel-per-pixel basis (pixel area ~4 km^2^) from:

$$m=\frac{4}{3}\frac{{\rho }_{ice}{r}_{e}\tau }{{Q}_{ext}}$$

where *ρ*_*ice*_ is the bulk density of ice (taken as 900 kg m^−3^). Total ice mass was computed from:

$${M}_{T}=\mathop{\sum }\limits_{i}^{{n}_{j}}\,{m}_{i}\,{A}_{i}$$

where *i* is the pixel number of area *A*_*i*_ and *n*_*j*_ is the total number of pixels for each hour, *j*. The area of analysis extends from 102 °E to 106 °E and 8 °S to 5.5 °S. Within this large area there are other convective systems generating ice not associated with the Anak Krakatau cloud. In order to reject these clouds from the total ice mass calculation, the Australian Bureau of Meteorology Volcanic Ash Advisories (VAAs) text message polygons were used to delineate the area of volcanic activity around Anak Krakatau. The VAA polygons can be accessed from ftp://ftp.bom.gov.au/anon/gen/vaac/ and are based on automated numerical model and observational analyses with occasional intervention by an expert meteorologist. They are issued at times appropriate for dissemination to interested consumers (e.g. aviation industry) and so the VAAs closest to the Himawari-8 acquisitions times were used (in most cases within 10–30 minutes). The VAAs used were not post-analysed, and while in the majority of cases they appear highly accurate, it was noticed that on some occasions differences between the VAA polygon and the satellite brightness temperature indication of the cloud, differed. No attempt was made to change the VAA polygons.

An error analysis was performed that included model errors (based on the sensitivity study described above), observational errors (accuracy of the brightness temperature measurements), errors due to including ice cloud not associated with Anak Krakatau, errors due to excluding Anak Krakatau ice clouds, mostly caused by cloud opacity too high for the retrieval scheme (i.e. Δ*T*’s ~0–1 K), and potential errors due to background convective activity within the VAA boundary but not necessarily associated with the Anak Krakatau cloud. To estimate this last source of error, the ice mass was calculated in a control area to the south of the volcano covering the region 105–105.9 °E and 7.8–6.8 °S. The mean ice mass found for this region was 0.16 × 10^9^ kg ±0.14 × 10^9^ kg compared with a mean value of 3.14 ± 2.02 × 10^9^ kg for the Anak Krakatau cloud. The hourly uncertainty on the ice cloud mass retrievals was found to be between ~10% and ~170% with a mean value of ~60%. A data file containing the hourly ice mass retrievals, mean mass loading, mean and median effective radius and total area used is included as Supplementary Data File 2.

The volcanic ice mass has also been computed using the level 2 cloud products derived from the measurements of the Moderate Resolution Imaging Spectroradiometer (MODIS) satellite instruments, on board the polar NASA-Terra and NASA-Aqua satellites (MOD06 and MYD06, respectively)^71^. Nine daytime products over the Anak Krakatau area have been collected covering the period from 22 to 28 December 2018 (Supplementary Table 2). From these data the cloud products of “Cloud Phase at 1-km resolution”, “Cloud Particle Effective Radius”, “Cloud Optical Thickness”, “Cloud Particle Effective Radius Relative Uncertainty”, “Cloud Optical Thickness Relative Uncertainty” and “Ice Extinction Coefficient Efficiency” have been extracted. For the Effective Radius and Cloud Optical Thickness products, the two channel retrievals using band 7 (2.1 μm) and 6 (1.6 μm) have been considered. All the products have been georeferenced and the ice mass loading computed on a pixel-per-pixel basis (pixel area 1 km^2^) by considering the same equation used for the Himawari-8 ice mass calculation with the same ice bulk density. Also the ice mass uncertainty has been computed in a pixel-per-pixel base from the Effective Radius and Cloud Optical Thickness relative uncertainties. The same VAA polygons were used to estimate the MODIS total ice mass for consistency with the Himawari-8 retrievals. The error associated with the total ice mass is the mean error computed in the same area.

### Cold-point and lapse rate tropopause

We have used the Global Navigation Satellite System (GNSS) Radio Occultation (RO) profiles^72^ from the COSMIC Data Analysis and Archive Center (CDAAC) to analyse the tropopause altitude in the area of the volcano. The climatological values were computed using all the profiles from 2006 to 2018 in a box of 5° latitude and 5° longitude around the volcano. The climatological Coldest Point Tropopause (CPT) altitude in December around the Anak Krakatau region is 17.29 km whereas the climatological Lapse Rate Tropopause (LRT) is 16.78 km with a standard deviation of 0.8 km. In January the CPT slightly rises to 17.63 km and the LRT to 17.01 km. In December 2018, before the Anak Krakatau eruption, the tropopause altitude was consistent with the climatological values (CPT 17.24 km and LRT 17.02 km) but the eruption changed the atmospheric conditions and the CPT and LRT altitudes diverged reaching respectively 18.7 km and 15.9 km in January 2019. The RO temperature profiles during the eruption, compared to the climatological temperature profile, show a warming of the upper troposphere (15.5 km – 17 km) with a peak amplitude of 4 K and a cooling of the lower stratosphere (18 km – 20.5 km) with amplitude up to −4 K. After the eruption, the stratospheric cooling persisted for at least 1 month. Only one RO was found (01:07 UTC on 28 December) in the area of the eruption and the cloud-top altitude computed by using the bending angle anomaly^73^ is 18.1 km suggesting a stratospheric cloud at least 1 km higher than the climatological tropopause.

### SO_2_ measurements

We compiled SO_2_ vertical columns from UV and IR polar orbiting sensors hosted by the SACS service^74^, with the assumption of a volcanic plume in the low stratospheric layer (except for IASI-A and B for which SO_2_ height retrieval is directly used^75^). The selection of SO_2_ pixels (considered for the mass loading) is applied using a threshold, except for TROPOMI for which the criteria is based on the ratio of SO_2_ measurement and its noise. For OMI and TROPOMI sensors, the estimate of SO_2_ mass loading is straight forward (considering surface of each footprint), while IR sensors and the composite of GOME2-A and B required a gridding to optimise the estimate of SO_2_ mass loading (grid of 0.25° × 0.25° and 0.5° × 0.5°, respectively).

### Lightning data analysis

Earth Networks Global Lightning Network (ENGLN) continuously measures lightning stroke occurrence time, location, type (IC and CG), polarity, and peak current, at over 1,600 ground-based stations around the world. ENGLN combines data from the Earth Networks Total Lightning Network (ENTLN) and the World Wide Lightning Detection Network (WWLLN). ENTLN is comprised of wideband sensors (5 kHz – 10 MHz) and uses time of arrival (TOA) techniques to detect both the IC and CG stroke signals^76^. ENTLN clusters stroke events (both IC and CG) occurring within spatial-temporal intervals of 0.7 seconds and 10 km into flashes. WWLLN is a network of lighting location sensors using time of group arrival (TOGA) techniques and operates at very low frequencies (3–30 kHz), which allows it to detect lightning from relatively large distances^77^. We considered all lightning flashes within a 100 km radius centred on Anak Krakatau in our analysis. To investigate the degree to which lightning from regional storms (independent of eruptive activity) affected our analysis, we reduced the search radius from 100 to 50 km and found that the number of recorded events during the time of observation reduced to 98.5% of the total. For a search radius of 30 km (roughly corresponding to the size of the plume umbrella; see Fig. 1), the number of events is 94% of the total. This confirms that the influence of storms concomitant to the eruption of Anak Krakatau was minimal and can hence be neglected for the scope of our analysis.

Different studies have proposed robust quantitative relationships between lightning flash rate, cloud-top height and peak thunderstorm updraft speed^34^. Lightning parameterizations based on cloud-top height are a commonly used method for predicting flash rate in global chemistry models^78^. In particular, Price and Rind^34^ proposed a lightning flash rate per minute, *F*, relationship of the following form to fit radar height, *H* (km), data:

$$F=a{H}^{b}$$

where *a* and *b* empirical parameters, with a ≈ 3.44 × 10^−5^ and b ≈ 4.9. We used such a relationship to fit satellite cloud-top heights (km) as a function of lightning flash rate (per 10 minute):

$$H=c{F}^{d}$$

where *c* and *d* are empirical parameters. Parameters obtained by best fitting the data gave $c=12.54\pm 0.45\,$ and $d=0.05\pm 0.008$ (Supplementary Fig. 2).

### Volcanic-CAPE analysis

To assess the convective instability of the environment prior to the convective phase of the Anak Krakatau eruption, we calculated the surface-based convective available potential energy (CAPE) for a sounding at Jakarta airport at 12:00 UTC on 22 December 2018. Figure 5a shows that the environment was conditionally unstable, but was not conducive to deep convection and severe thunderstorm activity (CAPE ~810 J/kg). However, if we consider that heat and moisture was being added to surface air parcels, via magma-seawater interactions at the volcano, then a significant increase in parcel buoyancy (CAPE) would be expected. We define the increase in CAPE due volcanic activity at the surface as ‘volcanic-CAPE’. We consider this effect by incrementally increasing surface parcel temperature and mixing ratio (Fig. 4b–d) and then computing the resulting parcel profiles using parcel theory. We find that only modest increases in heat (+8 °C) and moisture (+4 g/kg) are required to lift air parcels up to equilibrium levels close to the observed heights of the Anak Krakatau convective anvil (~16–17 km). These calculations indicate CAPE values consistent with extreme convection (5000–6000 J/kg). We repeated this analysis for all sounding profiles taken at Jakarta Airport during 22–28 December 2018 and found similar results to that shown in Fig. 5. All CAPE and parcel profile calculations were made using the Unidata MetPy package^79^. We note that the virtual temperature correction^80^ was not applied here as we did not have mixing ratio profiles from the Jakarta soundings for upper levels (>10 km). However, for large CAPE values (>4000 J/kg) this correction is expected to be small (relative errors <10%)^80^.

## Supplementary information

Supplementary Video 1.

Supplementary Dataset 1.

Supplementary Dataset 2.

Supplementary information

## Data Availability

Himawari-8 plume heights and ice mass loadings are available as Supplementary Data files. All other datasets generated and analysed during this study are available from the corresponding author upon reasonable request.

## Code availability

FPLUME source code is available upon request from A. F. or A. C.
