# Author Correction: Anak Krakatau triggers volcanic freezer in the upper troposphere

**DOI:** 10.1038/s41598-020-64152-8

**Published:** 2020-04-24

**Authors:** A. T. Prata, A. Folch, A. J. Prata, R. Biondi, H. Brenot, C. Cimarelli, S. Corradini, J. Lapierre, A. Costa

**Affiliations:** 1 Barcelona Supercomputing Center, Computer Applications in Science and Engineering, Barcelona, Spain; 2 AIRES Pty. Ltd., Mt Eliza, Victoria, Australia; 3 Visiting Professor, School of Electrical Engineering, Computing and Mathematical Sciences, Curtin University, Perth, Australia; 4 Università degli Studi di Padova, Dipartimento di Geoscienze, Padua, Italy; 5 Royal Belgian Institute for Space Aeronomy, Brussels, Belgium; 6 Department of Earth and Environmental Sciences, Ludwig-Maximilians-Universität, München, Germany; 7 Istituto Nazionale di Geofisica e Vulcanologia, Osservatorio Nazionale Terremoti, Rome, Italy; 8 Earth Networks Inc., Germantown, MD United States; 9 Istituto Nazionale di Geofisica e Vulcanologia Sezione di Bologna, Bologna, Italy

Correction to: *Scientific Reports* 10.1038/s41598-020-60465-w, published online 27 February 2020

A Supplementary Information File containing Figures S1–S5 and Tables S1–S2 was omitted from the original version of this Article. This has been corrected in the HTML version of the Article; the PDF version was correct at time of publication.

