# Supplementary material for: Anak Krakatau triggers volcanic freezer in the upper troposphere
Source: Sci Rep. 2020 Feb 27;10:3584. doi: 10.1038/s41598-020-60465-w (PMC7046738; doi:10.1038/s41598-020-60465-w)
Supplement: Supplementary file 4 — Supplementary information [file 41598_2020_60465_MOESM4_ESM.pdf]

Supplementary Information

Anak Krakatau triggers volcanic freezer in the upper troposphere

A. T. Prata[1*], A. Folch[1], A. J. Prata[2,3], R. Biondi[4], H. Brenot[5], C. Cimarelli[6], S. Corradini[7], J. Lapierre[8] & A. Costa[9]

[1] Barcelona Supercomputing Center, Computer Applications in Science and Engineering, Barcelona, Spain.

[2] AIRES Pty. Ltd., Mt Eliza, Victoria, Australia.

[3] Visiting Professor, School of Electrical Engineering, Computing and Mathematical Sciences, Curtin University, Perth, Australia.

[4] Università degli Studi di Padova, Dipartimento di Geoscienze, Padua, Italy.

[5] Royal Belgian Institute for Space Aeronomy, Brussels, Belgium.

[6] Department of Earth and Environmental Sciences, Ludwig-Maximilians-Universität München, Germany.

[7] Istituto Nazionale di Geofisica e Vulcanologia, Osservatorio Nazionale Terremoti, Rome, Italy.

[8] Earth Networks Inc., Germantown, MD, United States.

[9] Istituto Nazionale di Geofisica e Vulcanologia Sezione di Bologna, Bologna, Italy.

[*]email: andrew.prata@bsc.es

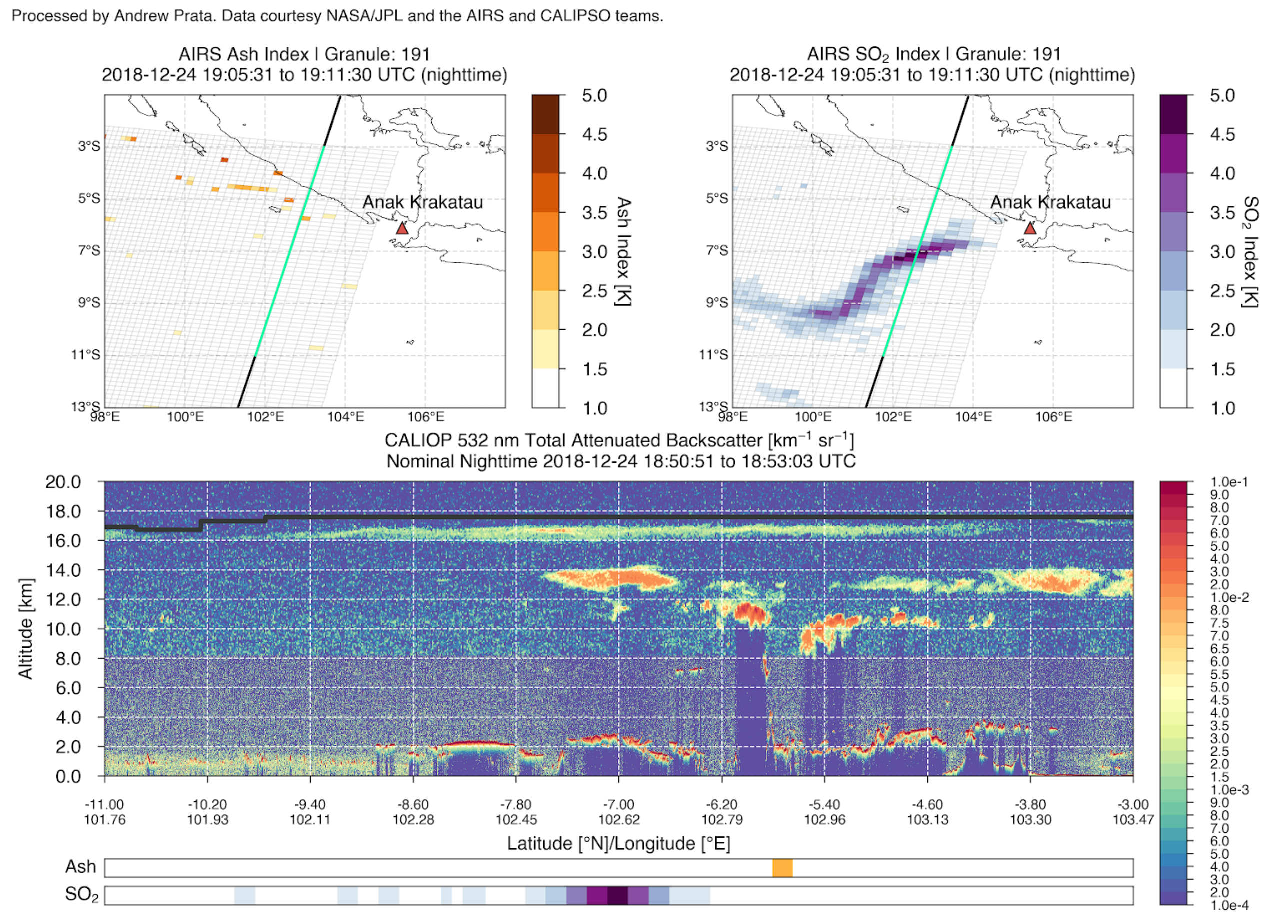

Supplementary Fig. 1 | CALIOP/AIRS analysis. Top left panel shows Atmospheric Infrared Sounder (AIRS)[1] ash detection index[2]. Black line with green highlight indicates Cloud-Aerosol Lidar with Orthogonal Polarization (CALIOP)[3] track. Top right panel same as top left but for AIRS SO_2 detection index[4]. Bottom panel shows CALIOP backscatter curtain with collocated AIRS pixels displayed as ash/SO_2 indicator bars beneath. Black line on curtain indicates tropopause. Methods used to produce this figure are described in Prata *et al.*[5].

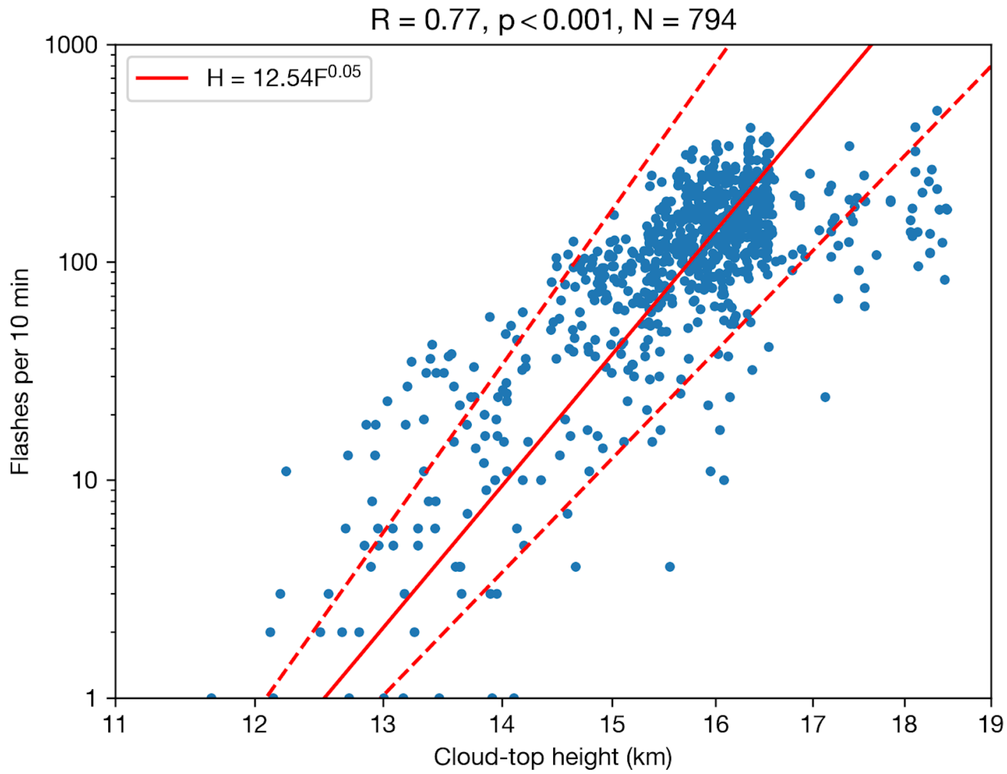

Supplementary Fig. 2 | Correlation between flash rate (F) and cloud-top height (H). Solid line indicates least squares fit using the equation, $H = cF^{d}$, where c and d are empirical constants (shown in legend). Dashed red lines indicate one standard deviation errors in c (± 0.450) and d (± 0.008). Correlation coefficient, R, and two-tailed p-value, p, were calculated by taking the Pearson's correlation coefficient of the two data sets (F and H) in logspace (correlation only uses non-zero values of F).

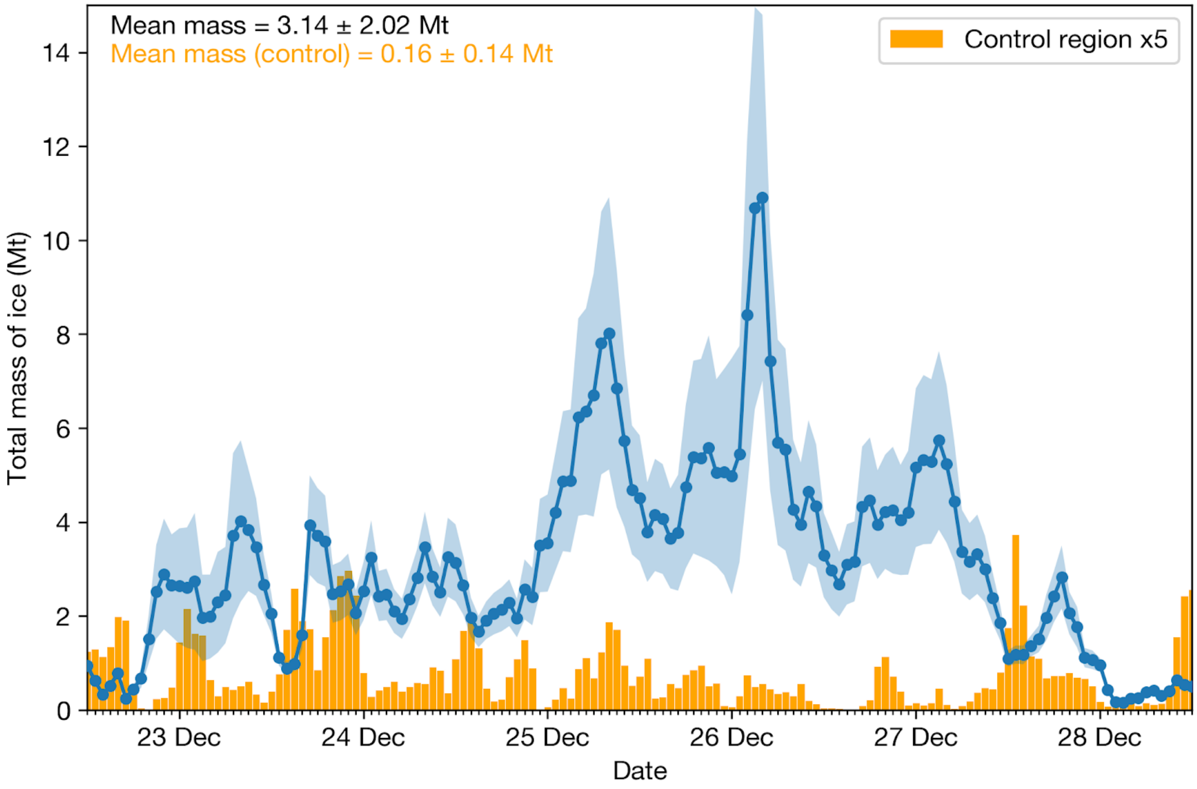

Supplementary Fig. 3 | Time series of Himawari-8 total ice mass. Solid line with dots indicates total ice mass (Mt) in the Anak Krakatau convective cloud from 12:00 UTC on 22 December 2018 to 12:00 UTC on 28 December 2018 at hourly intervals calculated from Himawari-8 infrared data (uncertainty indicated by shaded region). The total ice mass in the control region is shown by the orange-coloured bars and multiplied by 5 for clarity.

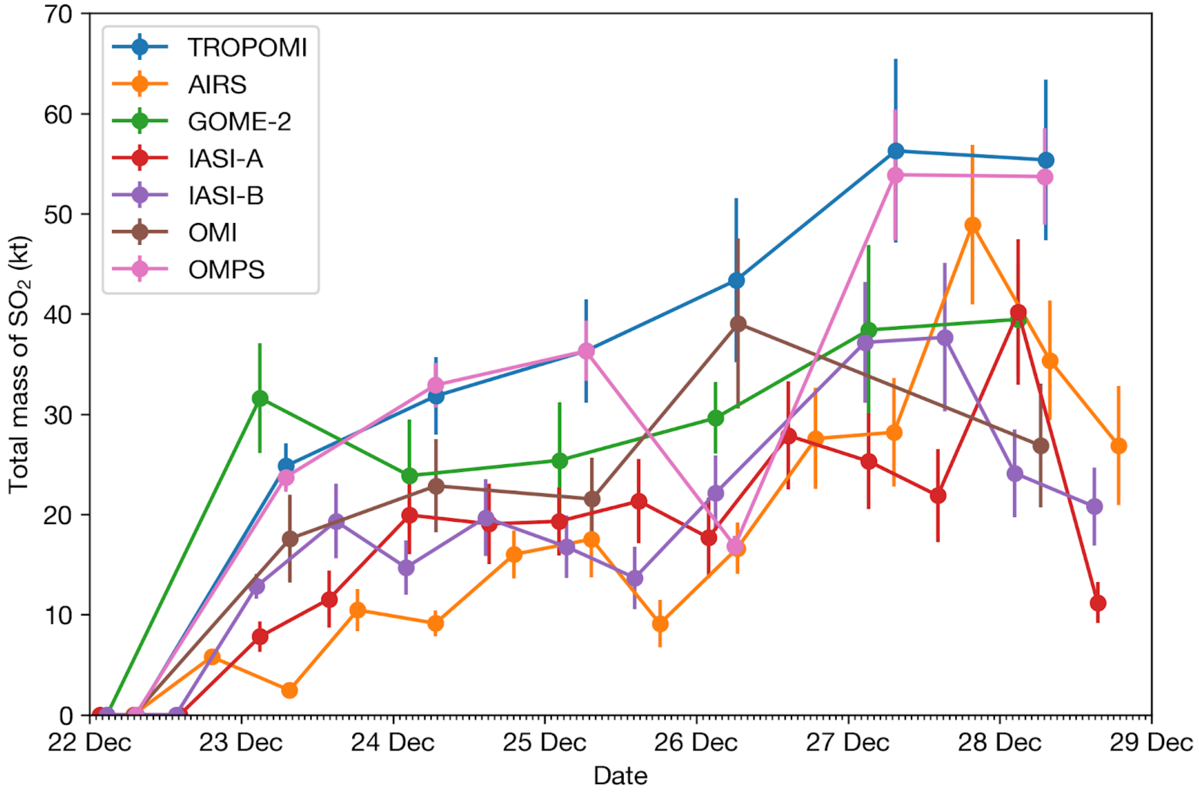

Supplementary Fig. 4 | Time series of SO_2 for the Anak Krakatau convective plume. Time-series indicates total mass of SO_2 for the following infrared and ultraviolet polar orbiting sensors taken from the SACS near-real time alert system[6]: TROPOspheric Monitoring Instrument (TROPOMI), Atmospheric Infrared Sounder (AIRS), Global Ozone Monitoring Experiment–2 (GOME-2), Infrared Atmospheric Sounding Interferometer-A, B (IASI-A, B), Ozone Monitoring Instrument (OMI) and Ozone Mapping Profiler Suite (OMPS). Errors in the total mass loadings were obtained by considering two types of Air Mass Factors (AMFs).

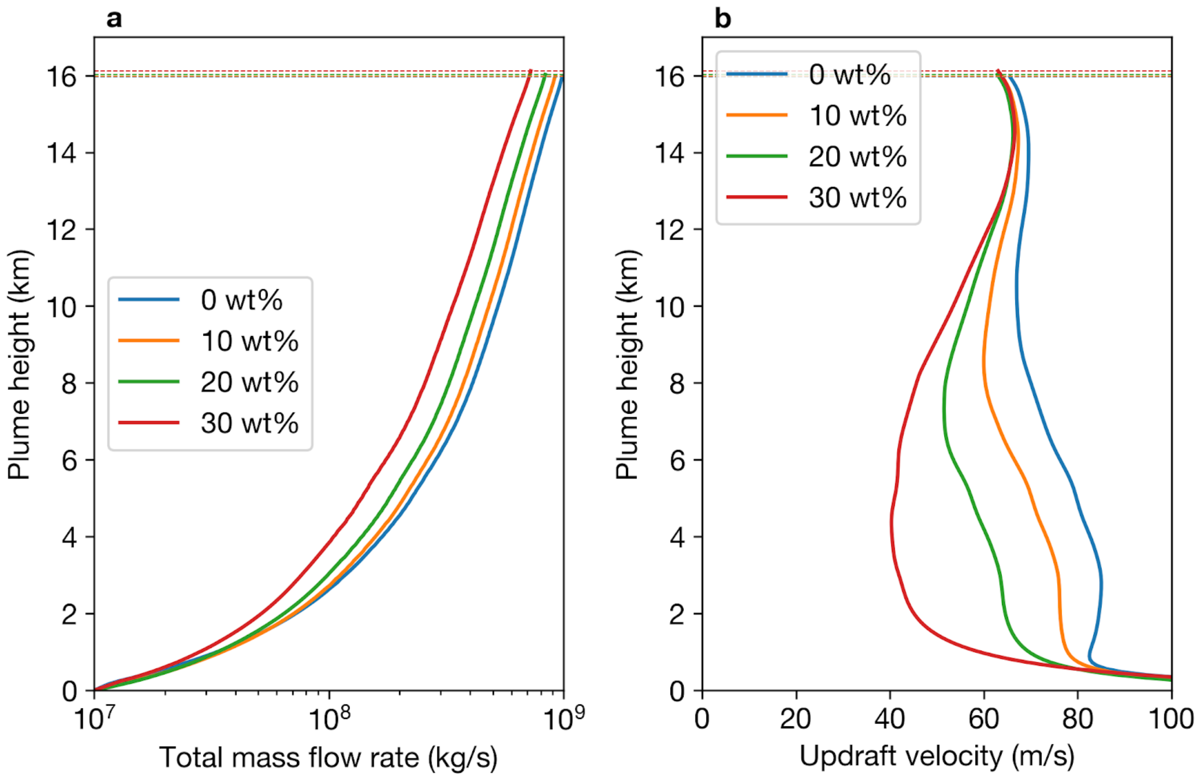

Supplementary Fig. 5 | FPLUME sensitivity study results. (a) Vertical profile of the total mass flow rate for different water fractions (0 wt%, 10 wt%, 20 wt% and 30 wt%). (**b)** Same as **(a)** but for updraft velocities. Horizontal dashed lines show neutral buoyancy level (NBL) for each simulation. For 0 wt%, 10 wt%, 20 %wt and 30 wt% the NBLs are 15.96 km, 15.98 km, 16.02 km and 16.12 km, respectively. Note that water fractions of 40 wt% and 50 wt% were considered, but these simulations resulted in column collapse.

Supplementary Table 1 | Volcanic input parameters for FPLUME sensitivity study. Note that the size distribution is computed automatically depending on column height and magma viscosity (10^6 Pa/s; see Costa *et al.*[7], for details).

Parameter	Value(s)
Vent elevation	0 m (sea level)
Mass flow rate	1×10^7 kg/s
Exit velocity	150 m/s
Exit temperature	1150 °C
Liquid water fractions	0, 10, 20, 30, 40 and 50 wt%
Gas (magma) water fraction	5 wt%

Supplementary Table 2 | MODIS level 2 cloud products[8] used to calculate ice mass loadings.

Satellite	Date (UTC)	Product
Terra	2018-12-23 03:05	MOD06
Terra	2018-12-24 03:45	MOD06
Aqua	2018-12-24 06:40	MYD06
Terra	2018-12-25 02:50	MOD06
Terra	2018-12-26 03:35	MOD06
Aqua	2018-12-26 06:25	MYD06
Aqua	2018-12-27 07:10	MYD06
Terra	2018-12-28 03:20	MOD06
Aqua	2018-12-28 06:15	MYD06

References

1. Chahine, M. T. *et al.* AIRS: Improving Weather Forecasting and Providing New Data on Greenhouse Gases. *Bull. Am. Meteorol. Soc.* **87**, 911–926 (2006).
2. Prata, A. T., Siems, S. T. & Manton, M. J. Quantification of volcanic cloud top heights and thicknesses using A-train observations for the 2008 Chaitén eruption. *J. Geophys. Res. Atmospheres* **120**, 2928–2950 (2015).
3. Winker, D. M. *et al.* Overview of the CALIPSO Mission and CALIOP Data Processing Algorithms. *J. Atmospheric Ocean. Technol.* **26**, 2310–2323 (2009).
4. Hoffmann, L., Griessbach, S. & Meyer, C. I. Volcanic emissions from AIRS observations: detection methods, case study, and statistical analysis. in (eds. Comerón, A. et al.) 924214 (2014). doi:10.1117/12.2066326.
5. Prata, A. T., Young, S. A., Siems, S. T. & Manton, M. J. Lidar ratios of stratospheric volcanic ash and sulfate aerosols retrieved from CALIOP measurements. *Atmospheric Chem. Phys.* **17**, 8599–8618 (2017).
6. Brenot, H. *et al.* Support to Aviation Control Service (SACS): an online service for near-real-time satellite monitoring of volcanic plumes. *Nat. Hazards Earth Syst. Sci.* **14**, 1099–1123 (2014).
7. Costa, A., Pioli, L. & Bonadonna, C. Assessing tephra total grain-size distribution: Insights from field data analysis. *Earth Planet. Sci. Lett.* **443**, 90–107 (2016).
8. Platnick, S. *et al.* MODIS cloud optical properties: User guide for the Collection 6 Level-2 MOD06/MYD06 product and associated Level-3 Datasets. *Version* **1**, 145 (2015).
